# Expression of **α**_v_**β**_6_integrin in oral leukoplakia

**DOI:** 10.1054/bjoc.1999.1130

**Published:** 2000-03-21

**Authors:** S Hamidi, T Salo, T Kainulainen, J Epstein, K Lerner, H Larjava

**Affiliations:** Faculty of Dentistry, University of British Columbia, 2199 Wesbrook Mall, Vancouver, BC, V6T-1Z3, Canada; Department of Diagnostic and Oral Medicine, Institute of Dentistry, University of Oulu, Aapistie 3, Oulu, 90220, Finland; Faculty of Dentistry, British Columbia Cancer Agency, Vancouver General Hospital, Vancouver, BC, V6T-1Z3, Canada

**Keywords:** integrins, leukoplakia, lichen, squamous cell carcinoma

## Abstract

The distribution of α_v_β_6_integrin was examined in oral leukoplakia, lichen planus and squamous cell carcinomas using immunohistochemistry. Controls included oral mucosal wounds, chronically inflamed and normal oral mucosa. Integrins β_1_, β_3_, β_4_, β_5_, fibronectin and tenascin were also studied. The integrin α_v_β_6_was highly expressed throughout the whole lesion of 90% of the squamous cell carcinomas but was not present in any of the normal specimens. α_v_β_6_integrin was also expressed in 41% of the leukoplakia specimens, and 85% of the lichen planus samples, but in none of the tissues with inflammatory hyperplasia or chronic inflammation. The expression of β1 integrins was localized in the basal layer, and that of the β_4_at the cell surface facing the basement membrane of all specimens. The integrins β_3_and β_5_were absent from all normal and leukoplakia specimens. Fibronectin and tenascin were present in the connective tissue underneath the epithelium of all the sections, and their expression was similar in both α_v_β_6_-positive and α_v_β_6_-negative tissues. A group of 28 leukoplakia patients were followed 1–4 years after first diagnosis. In this group, initially α_v_β_6_integrin-positive leukoplakia specimens had high tendency for disease progression while α_v_β_6_-negative specimens did not progress. These results suggest that the expression of α_v_β_6_integrin could be associated in the malignant transformation of oral leukoplakias. © 2000 Cancer Research Campaign


*British Journal of Cancer* (2000) **82**(8), 1433–1440
© 2000 Cancer Research Campaign
DOI: 10.1054/ bjoc.1999.1130, available online at http://www.idealibrary.com on IDEAL®

### Expression of $\alpha_v\beta_6$ integrin in oral leukoplakia

**S Hamidi[1], T Salo[2], T Kainulainen[3], J Epstein[3], K Lerner[3] and H Larjava[1]**

[1]Faculty of Dentistry, University of British Columbia, 2199 Wesbrook Mall, Vancouver, BC, V6T-1Z3, Canada; [2]Department of Diagnostic and Oral Medicine, Institute of Dentistry, University of Oulu, Aapistie 3, 90220 Oulu, Finland; [3]Faculty of Dentistry, British Columbia Cancer Agency, Vancouver General Hospital, Vancouver, BC V6T-1Z3, Canada

**Summary** The distribution of $\alpha_v\beta_6$ integrin was examined in oral leukoplakia, lichen planus and squamous cell carcinomas using immunohistochemistry. Controls included oral mucosal wounds, chronically inflamed and normal oral mucosa. Integrins $\beta_1$, $\beta_3$, $\beta_4$, $\beta_5$, fibronectin and tenascin were also studied. The integrin $\alpha_v\beta_6$ was highly expressed throughout the whole lesion of 90% of the squamous cell carcinomas but was not present in any of the normal specimens. $\alpha_v\beta_6$ integrin was also expressed in 41% of the leukoplakia specimens, and 85% of the lichen planus samples, but in none of the tissues with inflammatory hyperplasia or chronic inflammation. The expression of β1 integrins was localized in the basal layer, and that of the $\beta_4$ at the cell surface facing the basement membrane of all specimens. The integrins $\beta_3$ and $\beta_5$ were absent from all normal and leukoplakia specimens. Fibronectin and tenascin were present in the connective tissue underneath the epithelium of all the sections, and their expression was similar in both $\alpha_v\beta_6$-positive and $\alpha_v\beta_6$-negative tissues. A group of 28 leukoplakia patients were followed 1–4 years after first diagnosis. In this group, initially $\alpha_v\beta_6$ integrin-positive leukoplakia specimens had high tendency for disease progression while $\alpha_v\beta_6$-negative specimens did not progress. These results suggest that the expression of $\alpha_v\beta_6$ integrin could be associated in the malignant transformation of oral leukoplakias. © 2000 Cancer Research Campaign

**Keywords**: integrins; leukoplakia; lichen; squamous cell carcinoma

Integrins are a family of cell surface receptors that mediate cell–cell and cell–extracellular matrix adhesion in various cell types including epithelial keratinocytes (Watt and Jones, 1993; Larjava et al, 1996). These receptors are heterodimeric transmembrane glycoproteins composed of an alpha (α) and beta (β) subunit. Currently 22 different α and eight β-subunits are known. These subunits can variously combine to form more than 22 different cell surface receptors that have distinct ligand binding specificities.

Normal skin and mucosal epithelium express several different integrins. In normal epithelium, $\alpha_6\beta_4$ integrin binds to laminin-5 of the anchoring filaments and serves as an integral component of the hemidesmosome (Stepp et al, 1990; Sonnenberg et al, 1991). $\alpha_2\beta_1$ and $\alpha_3\beta_1$ integrins are localized in basal epithelial cells. They are known to be involved in cell–cell binding and binding of various collagen types and laminin-5 respectively (Carter et al, 1990; Staquet et al, 1990). A few integrins are generally absent from normal epithelium. Basal keratinocytes do not normally express $\alpha_v\beta_3$ and $\alpha_v\beta_6$ integrins (Breuss et al, 1995; Haapasalmi et al, 1995). $\alpha_v\beta_5$ has been detected from the same areas of normal buccal mucosa (Adams and Watt, 1991). On the other hand, it has been also reported to be absent from normal gingival epithelium (Larjava et al, 1993). $\alpha_v\beta_6$ is an exclusively epithelial integrin that has been shown to bind to fibronectin and tenascin (Sheppard et al, 1990; Prieto et al, 1993). Its expression is restricted to only a few locations in healthy adult tissues in humans (Breuss et al, 1993). Expression of $\alpha_v\beta_6$ integrin is, however, induced during wound healing and in squamous cell carcinoma (SCC) (Breuss et al, 1995; Haapasalmi et al, 1996).

It is not known at which stage of transformation of oral epithelial cells to SCC the expression of $\alpha_v\beta_6$ integrin begins. Epithelial cells that are in the process of malignant transformation can be found in some of the oral leukoplakia lesions. Studies indicate that malignant transformation of leukoplakia occurs over a range from about 1% to as high as 17%, averaging 4–5% (Gupta et al, 1980; Silverman et al, 1984). We investigated, therefore, whether epithelial cells in oral leukoplakia express $\alpha_v\beta_6$ integrin and whether this change could be associated with the malignant transformation.

### MATERIALS AND METHODS

### Tissues

Oral biopsy specimens of 29 cases of leukoplakia (11 from the gingiva, nine from the buccal or alveolar mucosa and nine from the tongue mucosa), eight of lichen planus (buccal mucosa), and 11 of SCCs were included in this study. From the leukoplakia patients, 11 were either current or past smokers, nine were non-smokers and for nine cases smoking history was unavailable. The diagnosis of leukoplakia and lichen planus were based on clinical and histological criteria. The leukoplakia tissue specimens were histologically graded (dysplasia, hyperplasia, hyperkeratosis, inflammation, etc.) by two pathologists independently (Tables 1 and 2). In case of differing opinion, biopsy specimen was re-examined and discussed until a consensus was reached. For controls, 11 normal oral specimens (six from the buccal mucosa and five from the gingiva), three chronically inflamed and five hyperplastic gingival tissue biopsies were originally taken during surgical

*Received 5 January 1999*
*Revised 2 November 1999*
*Accepted 11 November 1999*
*Correspondence to:* H Larjava

**Table 1** Expression of different integrins, fibronectin and tenascin in oral precancers and squamous cell carcinoma

| | Staining intensity | | | | | | | | |
|---|---|---|---|---|---|---|---|---|---|
| | ***n*** | $\beta_1$ | $\beta_3$ | $\beta_4$ | $\beta_5$ | $\beta_6$ | $\alpha_v$ | **FN** | **TN** |
| **Leukoplakia** | 29 | | | | | | | | |
| Dysplasia | 22 | | | | | | | | |
| Mild | 15 | ++ | – | ++ | – | –/+ | –/+ | +++ | +++ |
| Moderate | 6 | ++ | – | ++ | – | –/+ | –/+ | +++ | +++ |
| Severe | 1 | ++ | – | ++ | – | –/+ | –/+ | +++ | +++ |
| Other types | 7 | ++ | – | ++ | – | –/+ | –/+ | +++ | +++ |
| **Lichen planus** | 8 | | | | | | | | |
| All types | 8 | ++ | – | ++ | – | + | + | ND | ND |
| **Squamous cell carcinoma** | | | | | | | | | |
| Grade I | 6 | ++ | ND | ++ | ND | ++ | ++ | ND | ND |
| Grade II | 3 | ++ | ND | ++ | ND | ++ | ++ | ND | ND |
| Grade III | 2 | ++ | ND | ++ | ND | ++ | ++ | ND | ND |
| **Controls** | 22 | | | | | | | | |
| Normal mucosa | 11 | ++ | – | ++ | – | – | – | +++ | +++ |
| 7-day-old wound | 3 | ++ | ND | ++ | ND | ++ | ++ | +++ | +++ |
| Hyperplasia | 5 | ++ | – | ++ | – | – | – | ND | ND |
| Chronic inflammation | 3 | ++ | – | ++ | – | – | – | ND | ND |

Leukoplakia was classified to dysplasia or others (see Table 2). (–) No staining; (–/+) some specimens positive, some negative (see Table 4); (+) positive cells; (++) intense staining in basal cell layer; (+++) strong staining in basal cell layer, sometimes suprabasal cells and/or connective tissue. FN, fibronectin; TN, tenascin; ND, not determined.

**Table 2** Immunolocalization of $\alpha_v$ and $\alpha_v\beta_6$ integrin in oral lesions

| **Clinical diagnosis** | **Pathological diagnosis** | $\alpha_v$ | $\beta_6$ | **INF°** |
|---|---|---|---|---|
| Leukoplakia | HK, PK | 2/2 | 2/2 | 2 |
| Leukoplakia | HP | 3/4 | 3/4 | 2 |
| Leukoplakia | AT | 1/1 | 1/1 | 3 |
| Leukoplakia | DP | 6/22 | 6/22 | 2 |
| | • mild | 3/15 | 3/15 | 2 |
| | • moderate | 2/6 | 2/6 | 2 |
| | • severe | 1/1 | 1/1 | 2 |
| Reticular lichen planus | lichen planus | 7/8 | 7/8 | 3 |
| Squamous cell carcinoma | SCC | 10/11 | 4/5 | 0–2 |
| Normal mucosa | normal | 0/11 | 0/11 | 0 |
| Hyperplasia | HP | 0/5 | 0/5 | 0–3 |
| Periodontitis | CINF | 0/5 | 0/5 | 3 |
| Healing wounds | 7-day-old wound | 3/3 | 3/3 | 1 |

Numbers in the first two columns indicate the number of positive/total specimens examined in each case. HK, hyperkeratosis; PK, parakeratosis; HP, hyperplasia; AT, atypia; DP, dysplasia; CIFN, chronic inflammation; SCC, squamous cell carcinoma. Degree of inflammation (INF°) was visually graded as from 0 to 3 (from no inflammatory cells to abundant infiltration). The prevalence of $\alpha_v$ and $\alpha_v\beta_6$ integrin expression in leukoplakia, SCC and lichen planus is significantly increased compared to controls (ANOVA, $P < 0.05$).

procedures necessary for treatment. The hyperplastic lesions were either idiopathic (one case), drug-induced, e.g. amlodipine bensylate (one case), or caused by irritation by dentures (three cases). Seven-day-old human mucosal wound specimens were obtained from a collection stored in the laboratory (Larjava et al, 1993) (Tables 1 and 2). All samples were obtained from the University of British Columbia, Canada, British Columbia Cancer Agency, Canada, or the University of Oulu, Oulu, Finland.

The follow-up data of all the leukoplakia patients were collected from British Columbia Cancer Agency, where the original leukoplakia tissue specimens were collected. The progression or improvement of the disease in the long-term follow-up was assessed by a clinician who was unaware of all the staining results. The most recent follow-up information for each patient (1–4 years after the diagnosis) was collected from 28 of the 29 patients. The clinical and pathological data from the time of the biopsy of each patient was compared to the data of the last two recent visits of the patients. Patients were followed at least once a year after the original biopsy. If the conditions had progressed (either in size, or transformation to SCC), it was recorded as a disease progression. If none of the above conditions was applicable, then the disease was recorded as either no change, improved, or resolved accordingly. These results were then used to calculate the sensitivity and specificity of the $\alpha_v\beta_6$ integrin staining as a possible prognostic

 *© 2000 Cancer Research Campaign*

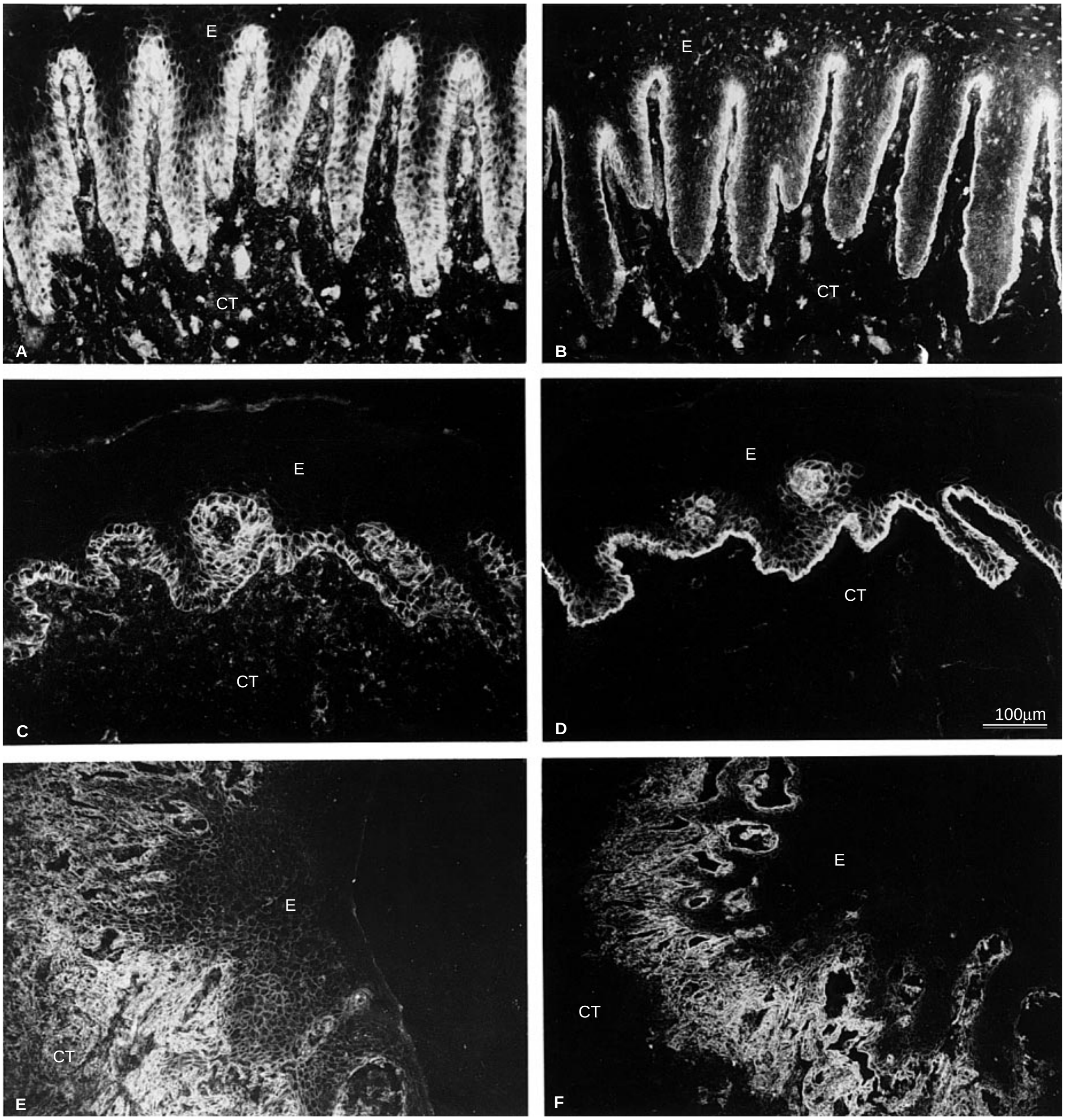

**Figure 1** Immunolocalization of $\beta_1$ and $\beta_4$ integrins in normal (**A, B**), leukoplakia (**C, D**) and squamous cell carcinoma (**E, F**) respectively. E, epithelium; CT, connective tissue; Bar = 100 µm test for disease progression. The most common treatments for the lesions were topical vitamin C cream (41% of patients) and beta carotene (31% of the patients).

### Antibodies

Monoclonal antibody to the $\beta_1$ integrin (mAb 13) subunit was a generous gift of Dr Kenneth Yamada, NIDR/NIH, and antibody to the $\alpha_v\beta_6$ integrin complex (E7P6) was a kind gift of Dr Dean Sheppard of Lung Biology Center, University of San Francisco. Monoclonal antibody to $\alpha_v$ integrin (L230) (Houghton et al, 1982) was purified from cell culture supernatant of hybridoma cells grown in our laboratory, and the antibodies against $\beta_4$ integrin (AA3; monoclonal), fibronectin and tenascin were purchased from Gibco-BRL (Gaithersburg, MD, USA). The monoclonal antibodies to $\alpha_v\beta_3$ integrin complex (mAb 1976), and $\alpha_v\beta_5$ integrin complex (mAb 1961) were purchased from Chemicon (Temecula, CA, USA).

### Immunofluorescence

Frozen sections (5 µm) were placed on glass slides which were treated with acetone containing 3-aminopropyl-triethoxy-silane (Tespa; Sigma Chemical Co., St Louis, MO, USA), and fixed briefly in chilled acetone (–20°C). Immunolocalization of integrins was performed as described previously (Larjava et al, 1993). Briefly, sections were washed with phosphate-buffered saline (PBS) containing 0.1% bovine serum albumin (BSA; Sigma

*© 2000 Cancer Research Campaign* 

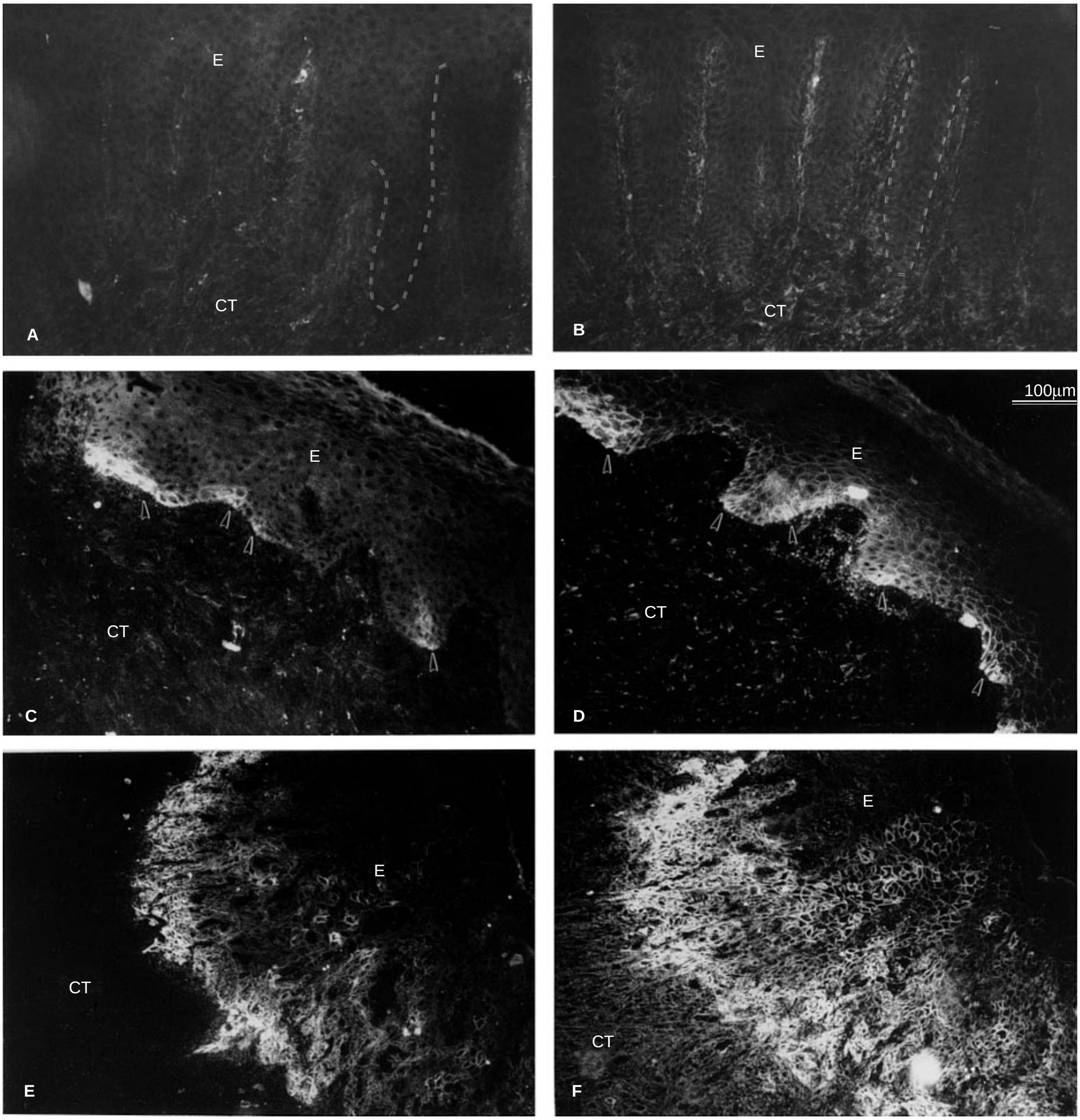

**Figure 2** Immunolocalization of $\alpha_v\beta_6$ integrin complex and $\alpha_v$ integrin subunit in normal (**A, B**), leukoplakia (**C, D**) and squamous cell carcinoma (**E, F**) respectively. Rete ridges are demonstrated using the dotted lines (**A, B**). The arrow heads point to the areas of cells that are reactive with antibodies to $\alpha_v\beta_6$ integrin complex (**C**) and $\alpha_v$ (**D**) integrin subunit. E, epithelium; CT, connective tissue; Bar = 100 μm

Chemical Co., St Louis, MO, USA) and incubated with optimally diluted primary antibodies in PBS/BSA in a humid chamber overnight. After rinsing, sections were incubated with affinity-purified rhodamine-conjugated secondary antibodies (1:50, Boehringer-Mannheim Biochemicals, Indianapolis, IN, USA) for 60 min. Sections were mounted using Krazy Glue (Borden Co. Ltd). Tissue specimens were stained with the primary antibodies as indicated (Table 1). Control stainings were performed using non-immune antibody or secondary antibody alone. Samples were examined using a Zeiss Axioskop 20 fluorescence microscope, and photographed using an MC 80 Zeiss microscope camera.

The intensity of the stainings was graded visually using a +/– scale. Specimens were classified as follows: (–) no staining was seen; (–/+) some sections were positive, and some negative (see Table 3); (+) some positive cells; (++) intense staining in the basal cell layer; and (+++) strong antibody staining in basal cell layer, sometimes suprabasal cells and/or connective tissue (stainings for fibronectin and tenascin). Staining with antibodies to integrins $\beta_1$ and $\beta_4$, fibronectin and tenascin produced uniform pattern in all specimens studied. Staining with antibodies recognizing $\alpha_v$ and $\alpha_v\beta_6$ integrins produced more variable results since some specimens were negative and some positive (–/+). Staining was considered positive and the intensity was scored if at least one rete ridge was reacted with the antibody.

 *© 2000 Cancer Research Campaign*

### RESULTS

### Localization of integrins and their binding molecules in normal mucosa

The integrins of $\beta_1$ and $\beta_4$ families were present in all normal tissues (Figure 1 and Table 1). $\beta_1$ integrins were localized at the periphery of the basal cells and in the connective tissue and endothelial cells (Figure 1A). $\beta_4$ integrin was localized at the basal surface of the basal keratinocytes (Figure 1B). Antibodies against either $\alpha_v\beta_6$ or $\alpha_v$ integrin were not reactive in normal specimens (Figure 2 A, B). Fibronectin and tenascin were both present in the connective tissue, especially in areas close to the basement membrane zone (not shown). Antibodies against $\beta_3$ or $\beta_5$ integrins were not reactive in normal oral mucosa (not shown) (Table 1).

### Localization of integrins and their binding molecules in leukoplakia

In leukoplakia, expression of $\beta_1$ and $\beta_4$ integrins resembled that of normal tissues (Figure 1 C, D). In some specimens, the expression of $\beta_1$ was found in several cell layers but often appeared somewhat reduced in the intensity (not shown). Forty-one per cent (12/29) of all the leukoplakia specimens expressed $\alpha_v\beta_6$ integrin. Twenty-seven per cent (6/22) of the dysplasia specimens expressed $\alpha_v\beta_6$ integrin, while 86% (6/7) of the other types (hyperkeratosis, hyperplasia and atypia) expressed it (Table 2). The expression was in most cases confined to the basal keratinocytes at the tip of the rete ridges. No or very little suprabasal expression was observed. Localization using antibodies to $\alpha_v$ or $\alpha_v\beta_6$ complex showed a similar distribution pattern (Figure 2 C, D). Epithelial cells of inflammatory, drug-induced or idiopathic hyperplasia or chronic inflammatory lesions (periodontitis) did not express $\alpha_v\beta_6$ integrin (Tables 1 and 2). None of the leukoplakia sections were reactive with $\beta_3$ or $\beta_5$ integrin antibodies (Table 1). Both fibronectin and tenascin were expressed underneath the oral epithelium of the leukoplakic tissues, at the area near the basement membrane zone similar to normal oral mucosa (Figure 3 A, B). Seven-day-old wounds were stained with antibodies to $\alpha_v$ and $\alpha_v\beta_6$ integrins as positive controls. In 7-day-old wounds $\alpha_v\beta_6$ integrin was present around basal cells covering the newly formed granulating tissue confirming our previous results (Haapasalmi et al, 1996) (Tables 1 and 2).

### Localization of integrins in lichen planus

The staining pattern of lichen planus specimens with antibodies against $\beta_1$ and $\beta_4$ integrin was similar to that of the normal tissues (Figure 4 A, B) except in some of the tissue specimens the staining was discontinuous in the basal cell layer, and patchy losses were also observed. In some areas of lichen planus, $\beta_4$ integrin was localized around the basal cells as we have observed before (Haapalainen et al, 1995). $\alpha_v\beta_6$ integrin was very strongly present around keratinocytes in basal and suprabasal cell layers of 85% (7/8) of all the lichen planus specimens studied. The staining pattern using antibodies to $\alpha_v\beta_6$ complex paralleled that of $\alpha_v$, however, the staining using $\alpha_v$ integrin antibody appeared to be relatively stronger (Figure 5 A, B). $\alpha_v\beta_6$ integrin was often seen in addition to the basal cell layer in several suprabasal cell layers (Figures 4C and 5B). Antibodies to $\beta_3$ and $\beta_5$ integrins were not reactive in lichen planus specimens (Figure 5 C, D).

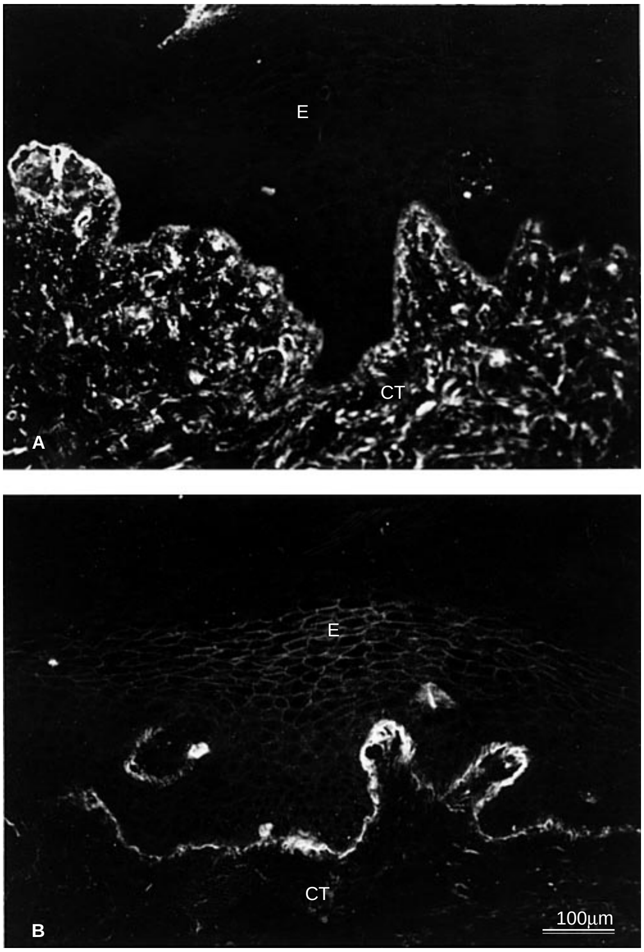

**Figure 3** Immunolocalization of fibronectin (**A**) and tenascin (**B**) in leukoplakia. E, epithelium; CT, connective tissue; Bar = 100 µm

### Localization of integrins in SCC

Several cell layers of squamous cell carcinoma lesions expressed $\beta_1$ and $\beta_4$ integrins (Figure 1 E, F). The staining pattern of these two integrins in the malignant tissues was more dominant and observed in more cell layers than that in the normal tissues. Antibodies to $\alpha_v$ and $\alpha_v\beta_6$ integrin complex were also strongly reactive in SCC (Figure 2 E, F). Tumour cells in several cell layers appeared to express these integrins. Eighty per cent (4/5) of the sections were positive when $\alpha_v\beta_6$ integrin antibody was used, and 90% (10/11) were positive when stained with $\alpha_v$ integrin antibody only.

### Follow-up data of the leukoplakia patients

The charts of all the leukoplakia patients were reviewed and their status at last follow-up to 1 year or more after the biopsies was studied. All the patients whose diseases had progressed expressed $\alpha_v\beta_6$ integrin. The five tissue specimens that expressed $\alpha_v$ $\beta_6$ integrin and showed disease progression represented two moderate dysplasias, and one severe dysplasia, one atypia which all progressed to SCC. One mild dysplasia progressed to recurrent dysplasia. Four leukoplakia specimens that were $\alpha_v\beta_6$ integrin-positive did not progress during the follow-up period. None of the

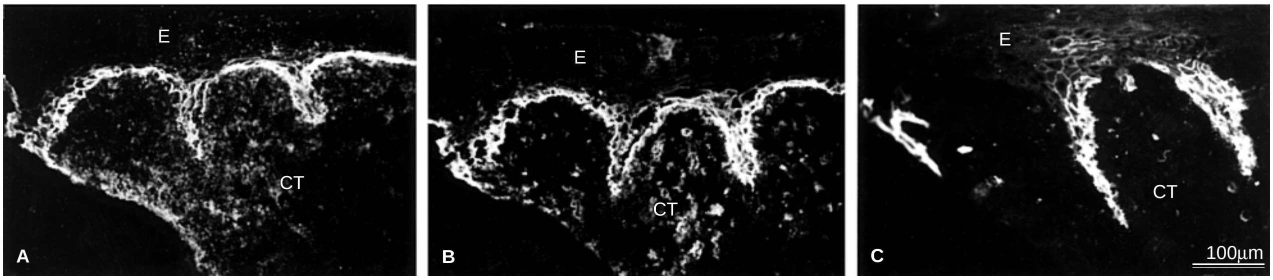

**Figure 4** Immunolocalization of $\beta_1$ (**A**), $\beta_4$ (**B**) and $\alpha_v\beta_6$ (**C**) integrins in parallel sections of oral lichen planus. E, epithelium; CT, connective tissue; Bar = 100 μm

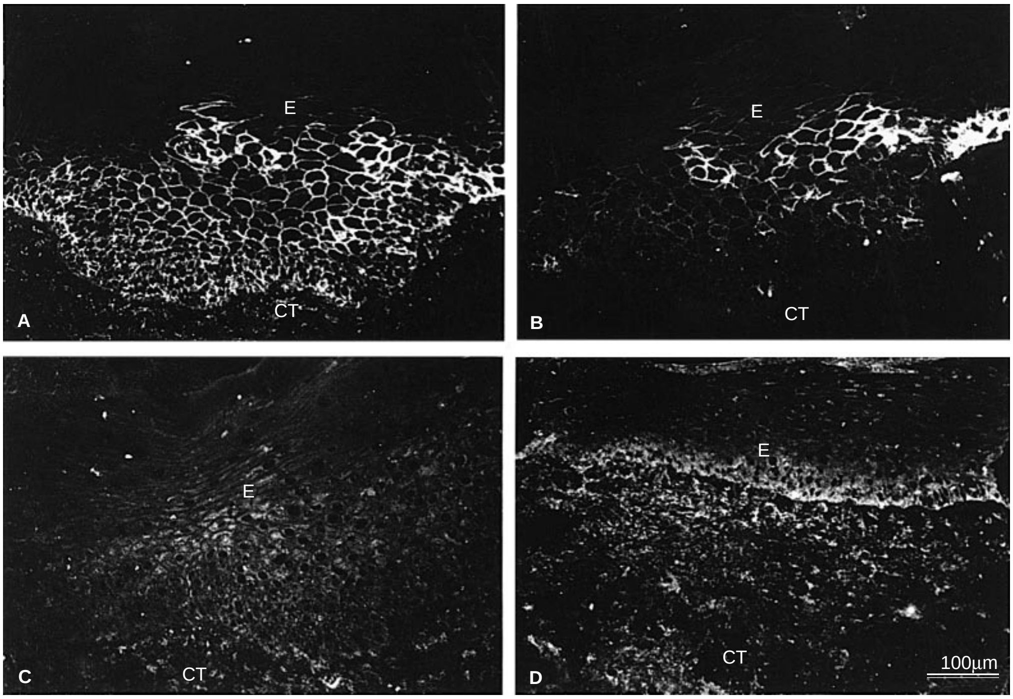

**Figure 5** Immunolocalization of $\alpha_v$ (**A**), $\alpha_v\beta_6$ (**B**), $\beta_3$ (**C**) and $\beta_5$ (**D**) integrins in lichen planus. E, epithelium; CT, connective tissue; Bar = 100 μm lesions ($n$ = 18) that did not initially express $\alpha_v\beta_6$ integrin progressed. Smoking history did not clearly correlate with the expression of $\alpha_v\beta_6$ integrin or disease progression. It should be kept in mind, however, that only a limited number of specimens from patients with positive smoking history were studied.

### DISCUSSION

The purpose of our study was to clarify whether epithelial cells in oral leukoplakia express $\alpha_v\beta_6$ integrin and whether this expression could be associated to malignant transformation of the lesions. Oral leukoplakia is a premalignant lesion that has potential to undergo malignant transformation (WHO, 1997). As high as 17% of the lesions may progress to malignant lesions of the oral cavity (Gupta et al, 1980; Silverman et al, 1984). $\alpha_v\beta_6$ is an exclusively epithelial integrin that is able to bind fibronectin and tenascin in the extracellular matrix (Sheppard et al, 1990; Prieto et al, 1993). Expression of $\alpha_v\beta_6$ integrin is induced during tumorigenesis and epithelial repair (Breuss et al, 1995; Clark et al, 1996; Haapasalmi et al, 1996). It has previously been shown that $\alpha_v\beta_6$ integrin is strongly expressed in SCCs of oral cavity (Breuss et al, 1995; Jones et al, 1997). Many of the cigarette smokers who develop lung cancer, express $\alpha_v\beta_6$ integrin in the proximal airway epithelium (Liebert et al, 1994).

In our study, 40% of the leukoplakia specimens expressed $\alpha_v\beta_6$ integrin. We were also able to show that those lesions that progressed during the follow-up period were $\alpha_v\beta_6$-positive although the material was relatively small. None of the initially $\alpha_v\beta_6$-negative leukoplakia progressed over time suggesting that a negative immunofluorescence finding for $\alpha_v\beta_6$ integrin could be used as a marker for non-progressive lesions. Since the portion of leukoplakia specimens that expressed $\alpha_v\beta_6$ is much higher than the reported rate of malignancy (maximally 17%), many of the positive lesions are not likely to be progressive. Several oncoproteins such as p53 and p16 have been tested as possible markers for malignant progression (Gallo et al, 1997). No single marker seems to be able to predict malignant transformation (Gallo et al, 1997). It remains to be shown whether the expression of $\alpha_v\beta_6$ integrin

 *© 2000 Cancer Research Campaign*

combined with other possible markers could be valuable in predicting malignant transformation of oral leukoplakia.

In addition to malignant transformation, there must be alternative explanations why so many leukoplakia specimens express $\alpha_v\beta_6$ integrin. One possible explanation is mechanical irritation or trauma that may be associated with leukoplakia, in which case induced $\alpha_v\beta_6$ integrin expression could be associated to epithelial repair. Subclinical inflammation is also reported to be associated with the induction of $\alpha_v\beta_6$ integrin expression in the lungs (Breuss et al, 1995). Epithelial cells in chronically inflamed oral mucosa appear $\alpha_v\beta_6$-negative, however, suggesting that inflammation alone is not sufficient to induce $\alpha_v\beta_6$ integrin expression (Haapasalmi et al, 1995). Furthermore, we observed that epithelial cells in most of the lichen planus specimens also expressed $\alpha_v\beta_6$ integrin. The frequency of malignant change in lichen planus ranges from 0.4 to 3.3% (Scully et al, 1998). It is likely therefore that expression of $\alpha_v\beta_6$ integrin in lichen has no association with malignant transformation. It is possible that the induced $\alpha_v\beta_6$ integrin expression could be associated to certain type of inflammatory reaction, such as predominance of lymphocytes in lichen planus. Alternatively, epithelial cell phenotypes that express $\alpha_v\beta_6$ integrin may be altered and play a role of controlling epithelial driven inflammation. Inactivation of $\beta_6$ integrin gene causes infiltration of macrophages into the skin and accumulation of lymphocytes around conducting airways in the lungs (Huang et al, 1996). Adding $\beta_6$ integrin gene back to alveolar type II cells and bronchiolar epithelial cells in $\beta_6$ knockout mice appears to reverse lung inflammation, suggesting that $\alpha_v\beta_6$ integrin may have a down-regulatory effect on pulmonary inflammation (Huang et al, 1998). Interestingly, it has been recently reported that $\alpha_v\beta_6$ integrin can bind and activate transforming growth factor $\beta_1$ (TGF-$\beta_1$) (Munger et al, 1999). This activation mechanism may have implications in cancer since overexpression of TGF-$\beta_1$ by epithelial cells enhances malignant progression rate and phenotype and induces high incidence of particularly malignant fibroblastoid spindle cell carcinomas (Cui et al, 1996).

It is likely that the induction of $\alpha_v\beta_6$ integrin could happen via alternative routes in oral leukoplakia specimens. In some specimens, this induction appears to be associated to the inflammatory reaction or tissue repair as discussed above. In the lesions that progressed to SCC, the induction of $\alpha_v\beta_6$ integrin expression is likely be linked to malignant transformation as a necessary but not sufficient prerequisite. Most cell lines derived from squamous cell carcinoma tumours express $\alpha_v\beta_6$ integrin (Koivisto et al, 2000). Colon carcinoma cell lines also frequently express $\alpha_v\beta_6$ integrin (Agrez et al, 1996). Heterologous expression of $\alpha_v\beta_6$ integrin in colon carcinoma cell line is associated to enhanced tumour growth (Agrez et al, 1994) which effect appears to be mediated through the cytoplasmic tail of $\beta_6$ integrin. In addition to its growth regulatory role, this unique cytoplasmic tail may also regulate signalling of gelatinase B (Niu et al, 1998). It is possible, therefore, that $\alpha_v\beta_6$ integrin could have multiple roles in malignant transformation, including cell adhesion and migration, regulation of cell growth and inflammation, and signalling of matrix degradation.

In summary, 41% oral leukoplakia specimens express $\alpha_v\beta_6$ integrin that could be associated to epithelial repair, inflammation and malignant transformation. Expression of $\alpha_v\beta_6$ integrin appears to be necessary but not sufficient for malignant transformation and it may have multiple roles in tumour formation.

### ACKNOWLEDGEMENTS

The authors thank Dr Kenneth Yamada and Dr Dean Sheppard for providing the antibodies for the study. This study was supported by grants from the Medical Research Council of Canada and the British Columbia Health Foundation.

### REFERENCES

Adams JC and Watt FM (1991) Expression of $\beta_1$, $\beta_3$, $\beta_4$, $\beta_5$ integrins by human epidermal keratinocytes and non-differentiating keratinocytes. *J Cell Biol* **115**: 829–841

Agrez MV, Bates RC, Mitchell D, Wilson N, Ferguson N, Anseline P and Sheppard D (1996) Multiplicity of fibronectin-binding $\alpha_v$ integrin receptors in colorectal cancer. *Br J Cancer* **73**: 887–892

Beck JD (1995) Issues in assessment of diagnostic tests and risk for periodontal diseases. *Periodontol 2000* **7**: 100–108

Breuss JM, Gillett N, Lu L, Sheppard D and Pytela R (1993) Restricted distribution of integrin $\beta_6$ mRNA in primate epithelial tissues. *J Histochem Cytochem* **41**: 1521–1527

Breuss JM, Gallo J, DeLisser HM, Klimanskaya IV, Folkesson HG, Pittet JF, Nishimura SL, Aldape K, Landers DV, Carpenter W, Gillett N, Sheppard D, Matthay MA, Albelda SM, Kramer RH and Pytela R (1995) Expression of the $\beta_6$ integrin subunit in development, neoplasia and tissue repair suggests a role in epithelial remodeling. *J Cell Sci* **108**: 2241–2251

Carter WG, Wayner EA, Bouchard TS and Kaur P (1990) The role of integrins $\alpha_2\beta_1$ and $\alpha_3\beta_1$ in cell–cell and cell–substrate adhesion of human epidermal cells. *J Cell Biol* **110**: 1387–1404

Clark RAF, Ashcroft GS, Spencer MJ, Larjava H and Ferguson MWJ (1996) Reepithelialization of normal human excisional wounds is associated with a switch from $\alpha_v\beta_5$ to $\alpha_v\beta_6$ integrins. *Br J Dermatol* **135**: 46–51

Cui W, Fowlis DJ, Bryson S, Duffie E, Ireland H, Balmain A and Akhurst RJ (1996) TGF$\beta_1$ inhibits the formation of benign skin tumors, but enhances progression to invasive spindle carcinomas in transgenic mice. *Cell* **86**: 531–542

Damjanovich L, Albelda SM, Mette SA and Buck CA (1992) Distribution of integrin cell adhesion receptors in normal and malignant lung tissue. *Am J Respir Cell Mol Biol* **6**: 197–206

Downer CS, Watt FM and Speight PM (1993) Loss of $\alpha_6$ and $\beta_4$ integrin subunits coincides with loss of basement membrane components in oral squamous cell carcinoma. *J Pathol* **171**: 183–190

Feliciani C, Gupta AK and Sauder DN (1996) Keratinocytes and cytokine/growth factors. *Crit Rev Biol Med* **7**: 300–318

Gallo O, Santucci M and Franchi A (1997) Cumulative prognostic value of p16/CDKN2 and p53 oncoprotein expression in premalignant laryngeal lesions. *J Natl Cancer* Inst **15**: 1161–1163

Gui GPH, Wells CA, Browne PD, Yeomans P, Jordan S, Puddefoot JR, Vinson GP and Carpenter R (1995) Integrin expression in primary breast cancer and its relation to axillary nodal status. *Surgery* **117**: 102–108

Gupta PC, Mehta FS, Daftary DK, Pindborg JJ, Bhonsle RB, Jalnawalla PN, Sinor PN, Pitkar VK, Murti PR, Irani RR, Shah HT, Kadam PM, Iyer KS, Iyer HM, Hegde AK, Chandrashekar GK, Shiroff BC, Sahiar BE and Mehta MN (1980) Incidence rates of oral cancer and natural history of oral precancerous lesions in a 10-year follow-up study of Indian villagers. *Community Dent Oral Epidemiol* **8**: 287–233

Haapalainen T, Oksala O, Kallioinen M, Oikarinen A, Larjava H and Salo T (1995) Destruction of epithelial anchoring system in lichen planus. *J Invest Dermatol* **105**: 100–103

Haapasalmi K, Mäkelä M, Oksala O, Heino J, Yamada KM, Uitto VJ and Larjava H (1995) Expression of epithelial adhesion proteins and integrins in chronic inflammation. *Am J Pathol* **147**: 193–206

Haapasalmi K, Zhang K, Tonnesen M, Olerud J, Sheppard D, Salo T, Kramer R, Clark RAF, Uitto VJ and Larjava H (1996) Keratinocytes in human wounds express $\alpha_v\beta_6$ integrin. *J Invest Dermatol* **106**: 42–48

Houghton AN, Eisenger M, Albino AP, Cairncross JG and Old LJ (1982) Surface antigens of melanocytes and melanomas. Markers of melanocyte differentiation and melanoma subsets. *J Exp Med* **156**: 1755–1766

Huang XZ, Wu JF, Cass D, Erle DJ, Corry D, Young SG, Farese Jr RV and Sheppard D (1996) Inactivation of the integrin $\beta_6$ subunit gene reveals a role of epithelial integrins in regulating inflammation in the lungs and skin. *J Cell Biol* **133**: 921–928

© 2000 Cancer Research Campaign

Jensen HM, Chen I, DeVault MR and Lewis AE (1982) Angiogenesis induced by 'normal' human breast tissue: a probable marker for precancer. *Science* **218**: 293–295

Jones J, Watt FM and Speight PM (1997) Changes in the expression of $\alpha_v$ integrins in oral squamous cell carcinomas. *J Oral Path Med* **26**: 63–68

Kobayashi T and Kawakubo T (1994) Prospective investigation of tumor markers and risk assessment in early cancer screening. *Cancer* **73**: 1946–1953

Koretz K, Schlag P, Boumsell L and Möller P (1991) Expression of VLA-$\alpha_2$, VLA-$\alpha_6$, and VLA-$\beta_1$ chains in normal mucosa and adenomas of the colon, and in colon carcinomas and their liver metastases. *Am J Pathol* **138**: 741–750

Koivisto L, Grenman R, Heino J, Larjava H. Integrins $\alpha_5\beta_1$, $\alpha_v\beta_1$ and $\alpha_v\beta_6$ in squamous carcinoma cell spreading and migration on fibronectin. *Exp Cell Res* (in press)

Koukoulis GK, Virtanen I, Korhonen M, Laitinen L, Quaranta V and Gould VE (1991) Immunohistochemical localization of integrins in the normal, hyperplastic and neoplastic breast. *Am J Pathol* **139**: 787–799

Larjava H, Haapasalmi K, Salo T, Wiebe C and Uitto V-J (1996) Keratinocyte integrins in wound healing and chronic inflammation of the human periodontium. *Oral Diseases* **2**: 77–86

Larjava H, Salo T, Haapasalmi K, Kramer RH and Heino J (1993) Expression of integrins and basement membrane components by wound keratinocytes. *J Clin Invest* **92**: 1425–1435

Liebert M, Washington R, Stein J, Wedemeyer G and Grossman HB (1994) Expression of the VLA $\beta_1$ integrin family in bladder cancer. *Am J Pathol* **144**: 1016–1022

Morson BC (1985) Precancer and cancer in inflammatory bowel disease. *Pathology* **17**: 173–180

Munger JS, Huang X, Kawakatsu H, Griffiths MJ, Dalton SL, Wu J, Pittet JF, Kaminski N, Garat C, Matthay MA, Rifkin DB and Sheppard D (1999) The integrin $\alpha_v\beta_6$ binds and activates latent TGF-$\beta_1$: a mechanism for regulating pulmonary inflammation and fibrosis. *Cell* **96**: 319–328

Nigam AK, Savage FJ, Boulos PB, Stamp GWH, Liu D and Pignatelli M (1993) Loss of cell–cell and cell–matrix adhesion molecules in colorectal cancer. *Br J Cancer* **68**: 507–514

Palecek SP, Loftus JG, Ginsberg MH, Lauffenburger DA and Horwitz AF (1997) Integrin–ligand binding properties govern cell migration speed through cell-substratum adhesiveness. *Nature* **385**: 537–540

Peltonen J, Larjava H, Jaakkola S, Gralnick H, Akiyama SK, Yamada SS, Yamada KM and Uitto J (1989) Localization of integrin receptors for fibronectin, collagen, and laminin in human skin. *J Clin Invest* **84**: 1916–1923

Pignatelli M, Smith MEF and Bodmer WF (1990) Low expression of collagen receptors in moderate and poorly differentiated colorectal adenocarcinomas. *Br J Cancer* **61**: 636–638

Pignatelli M, Hanby AM and Stamp GWH (1991) Low expression of $\beta_1$, $\alpha_2$ and $\alpha_3$ subunits of VLA integrins in malignant mammary tumors. *J Pathol* **165**: 25–32

Prieto AL, Edelman GM and Crossin KL (1993) Multiple integrins mediate cell attachment to cytotactin/tenascin. *Proc Natl Acad Sci USA* **90**: 10154–10158

Ruoslahti E (1991) Integrins. *J Clin Invest* **87**: 1–5

Scully C, Beyli M, Ferreiro MC, Ficarra G, Gill Y, Griffiths M, Homstrup P, Mutlu S, Porter S and Wray D (1998) Update on oral lichen planus: etiopathogenesis and management. *Crit Rev Oral Biol Med* **9**: 86–122

Sheppard D (1996) Epithelial integrins. *Bio Essays* **18**: 655–660

Sheppard D, Rozzo C, Starr L, Quaranta V, Erle DJ and Pytela R (1990) Complete amino acid sequence of a novel integrin β subunit ($\beta_6$) identified in epithelial cells using the polymerase chain reaction. *J Biol Chem* **265**: 11502–11507

Silverman S Jr, Gorsky M and Lazada F (1984) Oral leukoplakia and malignant transformation. A follow-up study of 257 patients. *Cancer* **53**: 563–568

Sonnenberg A, Calafat J, Janssen H, Daams H, van der Raaij-Helmer LMH, Falcioni R, Kennel SJ, Aplin JD, Baker J, Loizidou M and Garrod D (1991) Integrin $\alpha_6\beta_4$ complex is located in hemidesmosomes, suggesting a major role in epidermal cell–basement membrane adhesion. *J Cell Biol* **113**: 907–917

Staquet MJ, Levarlet B, Dezutter-Dambuyant C, Schmitt D and Thivolet J (1990) Identification of specific human epithelial cell integrin receptors as VLA proteins. *Exp Cell Res* **187**: 277–283

Stepp MA, Spurr-Michaud S, Tisdale A, Elwell J and Gipson IK (1990) $\alpha_6\beta_4$ integrin heterodimer is a component of hemidesmosomes. *Proc Natl Acad Sci USA* **87**: 8970–8974

Watt FM and Jones PH (1993) Expression and function of the keratinocyte integrins. *Development Suppl*: 185–192

Weinacker A, Ferrando R, Elliott M, Hogg J, Balmes J and Sheppard D (1995) Distribution of integrins $\alpha_v\beta_6$ and $\alpha_9\beta_1$ and their known ligands, fibronectin and tenascin, in human airways. *Am J Respir Cell Mol Biol* **12**: 547–556

WHO (1997) *Histological Typing of Cancer and Precancer of Oral Mucosa*. Springer: New York.

Yamamoto T and Osaki T (1995) Characteristic cytokines generated by keratinocytes and mononuclear infiltrates in oral lichen planus. *J Invest Dermatol* **5**: 784–788

Yamamoto T, Osaki T, Yoneda K and Ueta E (1993) An immunological investigation on adult patients with primary herpes simplex virus-1 infection. *J Oral Pathol Med* **22**: 263–267

Yang GY and Shamsuddin AM (1996) Gal-GalNAc: a bio-marker of colon carcinogenesis. *Histol Histopathol* **11**: 801–806

Zambruno G, Marchisio PC, Marconi A, Vaschieri C, Melchiori A, Giannetti A and De Luca M (1995) Transforming growth factor-$\beta_1$ modulates $\beta_1$ and $\beta_5$ integrin receptors and induces the de novo expression of the $\alpha_v\beta_6$ heterodimer in normal keratinocytes: implications for wound healing. *J Cell Biol* **129**: 853–865

*© 2000 Cancer Research Campaign*
